# A Three-Year Follow-Up of a Patient With Large Vestibular Aqueduct Syndrome Who Underwent Bilateral Endolymphatic Duct Blockage Surgery

**DOI:** 10.7759/cureus.70827

**Published:** 2024-10-04

**Authors:** Nayu Yokoyama, Yoshiyuki Sasano, Shinya Ohira, Fumihiro Mochizuki, Manabu Komori

**Affiliations:** 1 Department of Otolaryngology, St. Marianna University School of Medicine, Kawasaki, JPN

**Keywords:** endolymphatic duct blockage surgery, endolymphatic hydrops, endolymphatic sac decompression surgery, large vestibular aqueduct syndrome, meniere's disease

## Abstract

Large vestibular aqueduct syndrome (LVAS) is a congenital malformation characterized by an abnormally large vestibular aqueduct, diagnosed primarily via CT scans. Patients with LVAS often experience progressive hearing loss and recurrent vertigo, with treatment strategies mirroring those for Meniere's disease. Traditional surgical interventions such as endolymphatic sac decompression (ESD) are common; however, the efficacy of endolymphatic duct blockage (EDB) as an alternative still remains under investigation.

We present the case of a female in her 20s who was diagnosed with Meniere’s disease after presenting with hearing loss and recurrent vertigo since the age of 17. Despite conservative treatments and tympanostomy tube insertion, her symptoms worsened, prompting surgical intervention. CT scans revealed bilaterally enlarged vestibular aqueducts, which suggested LVAS. The patient underwent EDB combined with ESD on both ears, starting with the more affected right ear. The surgical approach involved mastoidectomy, exposure of the enlarged endolymphatic duct, placement of a 4 mm titanium clip followed by drainage of the endolymphatic duct. Over a three-year follow-up, the patient experienced no recurrence of dizziness or tinnitus. However, hearing in the right ear gradually deteriorated, suggesting the necessity of future cochlear implant surgery. The patient has returned to work and maintained stable vertigo control post-bilateral surgery.

This case introduces a new surgical method of combining EDB with ESD. Three years post-surgery, the method has resulted in potentially stabilized hearing in the better ear while effectively managing vertigo. It may be a solution to the challenge of vertigo in LVAS. The combined EDB and ESD procedure shows possibility for vertigo management in LVAS patients, along with the potential to preserve hearing when performed before significant deterioration. Despite hearing loss progression in the initially worse ear, the patient's quality of life improved significantly, highlighting the procedure's viability as a treatment option.

## Introduction

Large vestibular aqueduct syndrome (LVAS) is a congenital inner ear malformation with an abnormally large vestibular aqueduct, first reported in 1978 [1]. The vestibular aqueduct is a bony canal that runs from the inner ear into the skull. It contains a fluid-filled tube called the endolymphatic duct, which flows into a sac called the endolymphatic sac.

The diagnosis is made by CT. When the vestibular aqueduct midpoint diameter is greater than 1.5 mm, or greater than 2 mm at the operculum on CT scans, or if the aqueduct is grossly malformed on CT scans and/or MRI, it is defined as LVAS [2, 3]. Symptoms of LVAS are highly variable. The main symptoms are progressive hearing loss and recurrent vertigo. Since its treatment follows that of Meniere's disease, according to the international guideline of Meniere's disease, the first step of treatment is patient education such as the reduction of salt, alcohol, and caffeine [4]. The second step of treatment is diuretics. In severe cases where conservative therapy has failed, surgical treatment such as endolymphatic sac decompression (ESD) is considered. Although the medical treatment of LVAS follows that of Meniere's disease, surgical methods for LVAS are still controversial [5].

In recent years, endolymphatic duct blockage (EDB) has been proposed as an alternative surgical treatment to ESD [6, 7]. Here, we report a case of a typical LVAS patient in whom we performed EDB on both ears individually. During our 3-year follow-up, there has been no recurrence of dizziness or tinnitus.

## Case presentation

History of present illness

**Figure 1:**
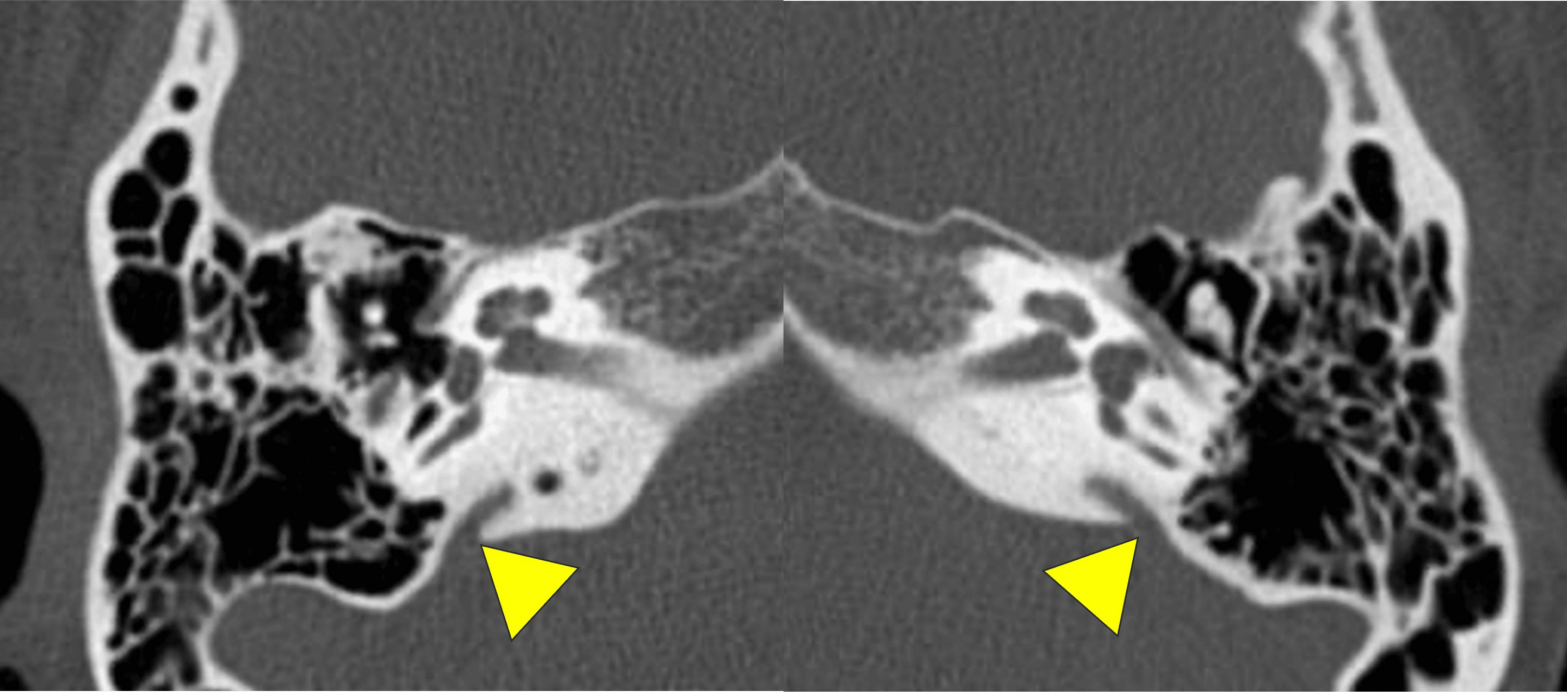
Pre-operation HRCT of the temporal bone (axial plane). Arrowheads indicate an enlarged vestibular aqueduct at the operculum. HRCT: High-Resolution Computed Tomography.

The patient is a woman in her twenties with chief complaints of hearing loss and recurrent vertigo attacks. She had been aware of hearing difficulties in her right ear for some time before visiting the previous hospital, but unfortunately, there is no detailed medical history. She first noticed bilateral hearing loss and vertigo at the age of 17 when she visited the previous hospital. She was initially treated conservatively for Meniere's disease. However, her symptoms worsened due to the COVID-19 outbreak, causing changes in lifestyle, increased stress, and sleeping disorders. Accordingly, tympanostomy tubes were inserted in both ears at the age of 19, which only temporarily restored her hearing. Despite treatment, her vertigo worsened, and she had to take time off from work, but still, her symptoms did not improve. Thus, she was referred to us for genetic testing, MRI of the inner ear, and surgery if necessary. At the first visit to our outpatient clinic, her external ear and external auditory canal showed no malformations. Tympanic membranes were intact in both ears, and tympanostomy tubes were observed in both ears. In addition, she already had hearing aids in both ears but had difficulty adjusting them due to frequent fluctuations in hearing. We performed pure-tone audiometry, speech audiometry, and vestibular exams. We also performed a high-resolution computed tomography (HRCT) of the temporal bone, which revealed an enlarged vestibular aqueduct (Figure 1), leading to the re-diagnosis of LVAS instead of Meniere's Disease. Her lack of improvement with medication indicated the necessity for surgery. Since her hearing was worse in the right ear, we decided to operate on that side.

Test findings at initial examination

**Figure 2:**
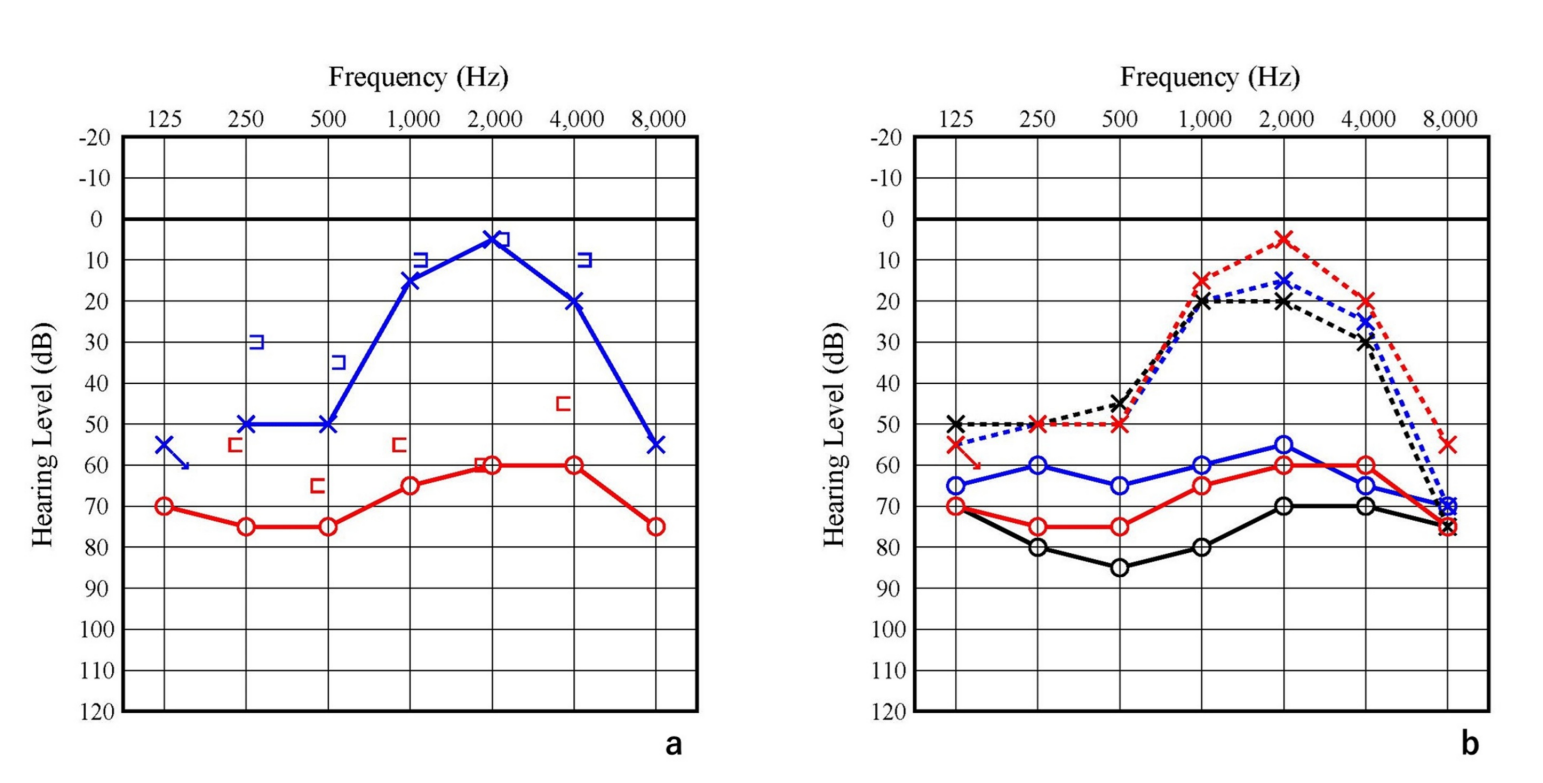
Pre-operation audiograms. 2a: Audiogram at first visit. An air-bone gap was recognized in places. 2b: Comparison of audiograms pre-operation. Red line: First visit; Blue line: Second visit; Black line: Last visit before operation.

At her first visit, her pure-tone audiometry averaged 68.8 dB in the right ear and 30 dB in the left ear using the AAO criteria [8], which is the average of the four frequency hearing levels from 250 Hz to 2000 Hz. Both sides showed higher thresholds in the low tone without an air-bone gap (Figure 2). The speech discrimination scores were 85% obtained at 100dB in the right ear and 95% obtained at 55dB in the left ear. The SLC26A4 mutations, described below, were not detected in the genetic test.

**Figure 3:**
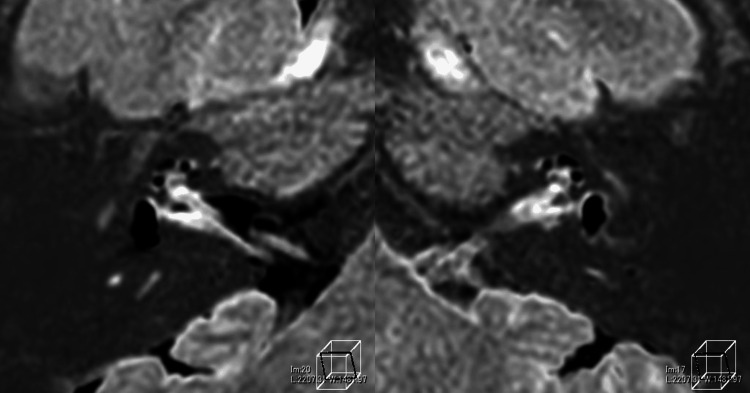
Pre-operation MRI (HYDROPS). Marked endolymphatic hydrops in the bilateral cochlea and vestibule were observed.

She underwent thorough vestibular function tests that showed left horizontal nystagmus in the sitting position, left dominant bilateral suppression in the caloric test, poor response in the right cervical vestibular evoked myogenic potential (cVEMP), negativity to glycerol VEMP, and normal response in video head impulse test (vHIT), which overall suggested that the patient has no vestibular inefficiency but hydrops instead. CT of the temporal bone showed vestibular aqueduct midpoint diameter enlargement over 1.5mm (R:2.4mm, L:2.8mm) on both sides, plus the diameter of both opercula were over 2mm (R:3.30, L:3.29mm) (Figure 1). This fulfills the criteria for LVAS. An MRI image using the HYDROPS method showed prominent endolymphatic space in both the vestibule and cochlea of both ears (Figure 3). Overall, the results indicated LVAS. 

Surgical procedure

**Figure 4:**
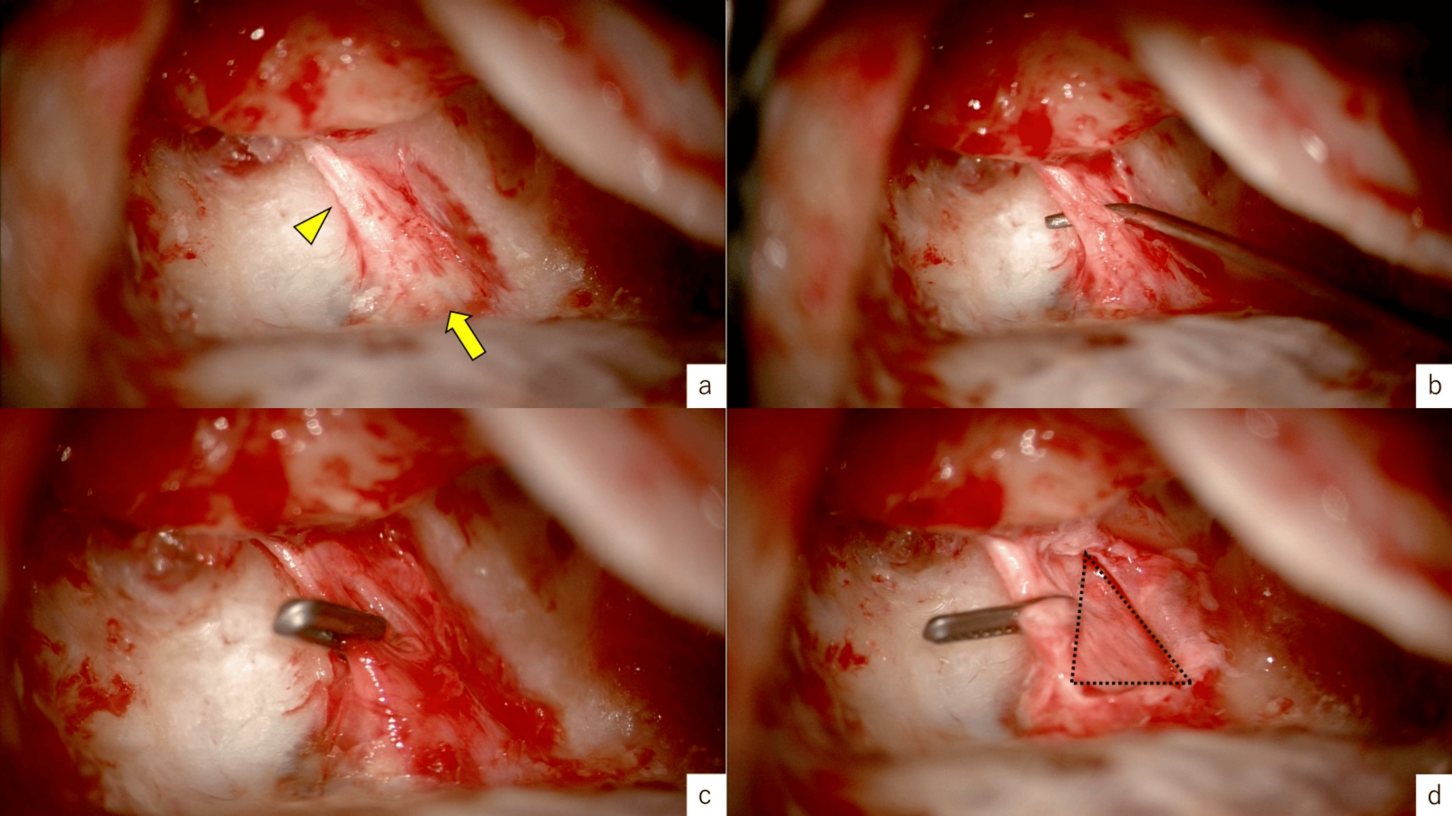
Surgery of the right ear. 4a: Vestibular aqueduct (arrowhead) and endolymphatic sac (arrow) identified. 4b: Dissecting vestibular aqueduct and endolymphatic sac with a pick. 4c: Clipping the vestibular aqueduct. 4d: Opening the endolymphatic sac. Dark cells (area inside dotted triangle) can be seen inside.

The surgery began with a mastoidectomy. We made a 4cm incision behind the right earlobe, then drilled the mastoid with a cutting burr, identified the sigmoid sinus, sinodural angle, and removed the surrounding air cells. After visualizing the lateral semicircular canal and the incus, the posterior semicircular canal was exposed, and the bony canal of the posterior fossa was thinned with a diamond burr. The sigmoid sinus was slightly protruding, which required some extra care. Finally, the enlarged endolymphatic duct was exposed and the endolymphatic sac was revealed. The sac was enlarged and inflated like a water balloon (Figure 4a). A pick was used to dissect the sac from the surrounding tissue and to create space for the insertion of the 4 mm titanium clip, typically used in neurosurgery for the clipping of the cerebral artery (Figure 4b, 4c). Finally, after clipping the endolymphatic duct, we incised the sac. Inside the sac, there were dark cells (Figure 4d).

After initial surgery

**Figure 5:**
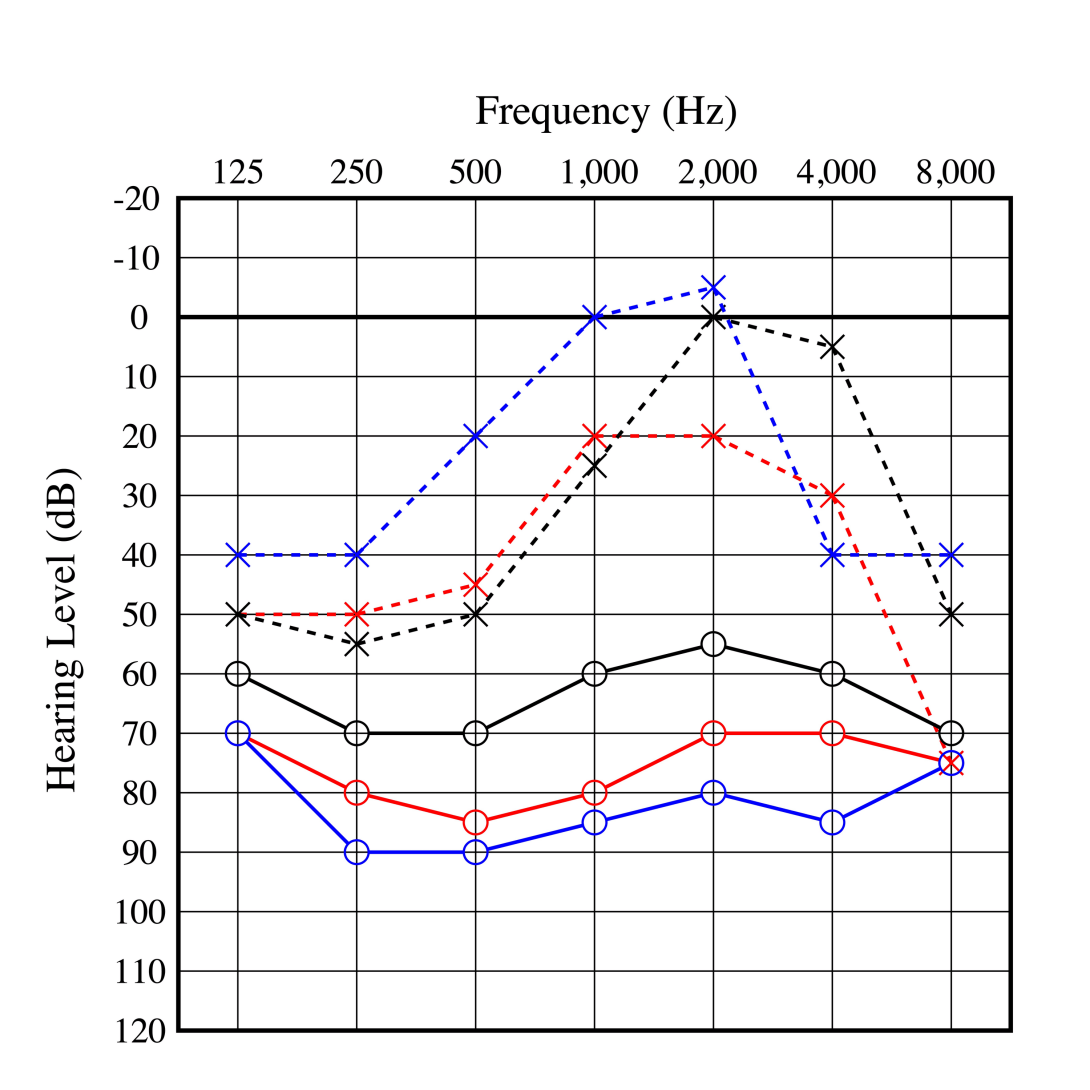
Pre and post-operation audiogram. The poor hearing ear showed temporary improvement after surgery but gradually worsened. The better hearing ear showed improvement after the surgery and has been maintained. Red line: Initial PTA; Black line: One week after bilateral ear operation; Blue line: Three years after bilateral ear operation. PTA: Pure tone audiometry.

Four months after the initial surgery, EDB was also performed on the other side. Around three years post-surgery, hearing in the right ear began to deteriorate. On the other hand, hearing in the left ear, which was the better-hearing ear, was maintained (Figure 5). Since the right ear has shown no recovery with close follow-up, a cochlear implant is planned for the future.

As for vertigo, there were some minor episodes after the single-ear surgery, but no episodes occurred after the bilateral surgery. The patient has been able to return to work and is currently working four days a week. The results of the postoperative vertigo equilibrium function tests for both ears were as follows: cVEMP in the right ear had improved, and the difference between the right and left ear had disappeared. The caloric test revealed that canal paresis in the right ear remained the same as before surgery, but canal paresis in the left ear had recovered, and vHIT remained normal as before the surgery. Thus, the vestibular function of the left ear, which was mild before surgery, recovered, but the vestibular function of the right ear, which was severe before surgery, did not recover. These results indicate that the vestibular function in LVAS is reversible.

**Figure 6:**
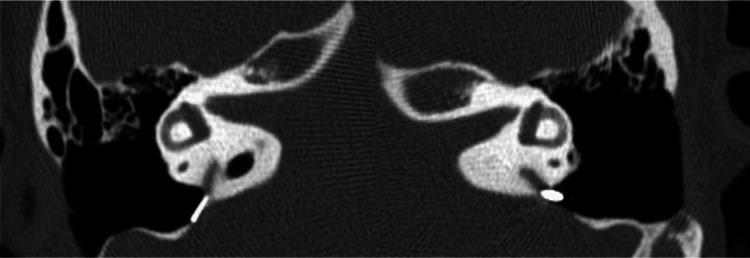
Postoperative CT. Postoperatively, the clips have been maintained without problems.

Postoperative CT confirms that the postoperative temporal bone is intact and that the clip is in good position (Figure 6).

## Discussion

There is still a lot we do not know about LVAS. However, a nationwide study of LVAS in Japan revealed data that we did not previously know. For example, hearing loss was present in 98.9% of 304 patients with LVAS, and 35.0% experienced vertigo [6]. Pure-tone audiometry showed profound hearing loss (PTA >91dB) in 316 (52.0%) of the 608 ears in the 304 patients tested, and asymmetric hearing loss, defined as >10dB, in 147 (48.4%) of the 304 patients. The mean PTA was 83.7dB (median, 91.3dB; interquartile range, 71.3-103.8dB), and the severity in PTA did not correlate with age. Hearing in LVAS patients with profound hearing loss or deafness did not change over time. Age 10 years or older, bilateral hearing loss, a history of head trauma, and Pendred syndrome were risk factors for fluctuating hearing loss and/or vertigo/dizziness.

Recent studies have shown an association of LVAS with the autosomal dominant mutation of the SLC26A4 gene located on the long arm of the 7th chromosome, especially in Asians, specifically in South Korea and Japan [9, 10]. This gene mutation is known to be involved in Pendred syndrome and autosomal recessive (AR) non-syndromic hereditary hearing loss (DFNB4) as well [11]. Pendred syndrome is inherited as an AR trait and requires mutations in both copies of the gene. However, according to an NIDCD study, there are patients with only one SLC26A4 gene mutation suggesting the possibility of a yet-to-be-discovered mutation [12]. As no genetic mutation of SLC26A4 was found in our case, we did not test further for Pendred syndrome. However, we may conduct another genetic search when genetic testing is expanded in the future.

Pathologically, it shows symptoms of fluctuating and progressive hearing loss along with recurrent vertigo. It is similar to Meniere's Disease, while it differs in that LVAS is more likely to have an air-bone gap in the bass region, and symptoms can be triggered by head trauma. There is yet no established treatment for LVAS. Although there is no consensus on conservative treatment, it often seems to follow that of Meniere's Disease as the clinical symptoms are similar. For example, initial dietary control and medication, subsequently recommending hearing aids and cochlear implants in cases of advanced hearing loss. As for vertigo, however, there are no aids nor medications except for those aimed to relieve accompanying symptoms such as nausea [7]. Thus, surgical options are the last resort. In Meniere's disease, ablative surgery such as surgical destruction of the labyrinth were performed in the past. The currently most recommended method is ESD, which was first reported in 1927 [13]. As the name suggests, ESD decompresses the endolymphatic sac by dissecting the sac.

Wilson and others performed endolymphatic sac obliteration in 7 ears of 6 patients in the late 1990s [14]. Throughout their follow-up period, which averaged 3.2 years, 6 out of 7 ears showed improved hearing. A relatively new method proposed in recent years is EDB. In this technique, the sac is not incised nor dissected from the posterior fossa dura. All the bone around the endolymphatic duct is dissected to identify the duct as much as possible, then the duct is blocked with two small titanium clips [15]. In 2015, Saliba et al. [6] reported a non-blinded randomized controlled trial comparing this technique against traditional ESD and found that 96.5% of the patients in the endolymphatic blockage group had achieved complete control of vertigo spells compared to 37.5% of the ESD group, with no significant difference between the preoperative and the postoperative hearing levels in both groups. Their study suggested that this novel technique may be better than ESD. In a follow-up study in 2016, with a larger group of patients, this same group reported a total absence of Meniere's attacks in 89.9% of the patients treated with this novel technique [15]. Further, they conducted a study on 11 LVAS patients who underwent EDB and evaluated their change in dizziness and hearing [7]. Accordingly, all 6 subjects who had preoperative dizziness showed improvement, and hearing was stabilized or improved in 82% of the cases, concluding that EDB is helpful for dizziness control and hearing improvement in LVAS patients.

In our case, we conducted the EDB method in combination with drainage. First, we clipped the endolymphatic duct, then further incised the endolymphatic sac. Although both ears showed postoperative hearing improvement, the ear with initially worse hearing gradually deteriorated, while the ear with initially better hearing remained stable. The reason for combining the two methods was based on the assumption that in LVAS, some pressure is transmitted to the inner ear through the large endolymphatic sac and the vestibular aqueduct, causing the disorder. As Issam Saliba et al. have hypothesized, there is increased pressure in the inner ear when endolymph secretion outweighs absorption [6]. If the patient were to make an incision without clipping first, a large pressure change would occur in the inner ear, which could result in hearing loss. Therefore, we thought that making the incision after clipping would also be useful to protect the inner ear. In our case, even when the clipping is insufficient, the incision prevents pressure from the endolymphatic sac from being transmitted to the inner ear. This has worked very well, and the dizziness has been almost completely controlled for three years post-operation. On the other hand, progression of hearing loss in the originally worse ear has been observed. This suggests that the cause of hearing loss in LVAS may not be limited to the enlarged endolymphatic sac and vestibular aqueduct but may also have some intrinsic causes.

Complications of the surgery are the same as for ESD, such as cerebrospinal fluid leakage, worsening tinnitus, vertigo, and hearing loss. Thus, if the symptoms are mild, we do not recommend surgery. Also, if the patient is too young, there is a risk that the temporal bone is still in the process of development. Therefore, we believe it is safe to perform the operation if the patient is over 10 years old. A significant advantage is that the surgery can be performed with the same surgical skills as for ESD, which makes it accessible.

Even if there is recurrence and worsening of hearing loss in the future, we believe that this clipping does not preclude cochlear implant surgery. Thus, our combination treatment of EDB and ESD is effective, especially for vertigo in LVAS, and it does not interfere with cochlear implant surgery, even if it is chosen in the future.

## Conclusions

We experienced a case of LVAS in which we performed combined EDB and ESD by both clipping and then draining the endolymphatic duct. The surgery was performed on each ear separately in two separate sessions. Three years after the second surgery, vertigo has completely resolved, but hearing in the originally poor ear is slowly deteriorating.

Although the pathogenesis of LVAS is still unknown, we believe that the control rate of this procedure combining EDB and ESD for vertigo attacks is relatively good. It has also been suggested that hearing deterioration may be prevented by performing the procedure before it progresses.
